# Nuclear pore complex protein RANBP2 and related SUMOylation in solid malignancies

**DOI:** 10.1016/j.gendis.2024.101407

**Published:** 2024-09-04

**Authors:** Xinning Yu, Huatao Wu, Zheng Wu, Yangzheng Lan, Wenjia Chen, Bingxuan Wu, Yu Deng, Jing Liu

**Affiliations:** a Department of General Surgery, The First Affiliated Hospital of Shantou University Medical College, Shantou, Guangdong 515041, China; b The Breast Center, Cancer Hospital of Shantou University Medical College, Shantou, Guangdong 515041, China; c Department of Physiology, Shantou University Medical College, Shantou, Guangdong 515041, China

**Keywords:** Cell cycle, Malignancy, Oncogenesis, RANBP2, SUMOylation

## Abstract

The growing interest in post-translational protein modification, particularly in SUMOylation, is driven by its crucial role in cell cycle regulation. SUMOylation affects various cell cycle regulators, including oncogenes, suggesting its relevance in cancer. SUMO E3 ligases are pivotal in this process, exhibiting diverse functionalities through structural domains and subcellular localizations. A less-explored SUMO E3 ligase, RANBP2, a component of the vertebrate nuclear pore complex, emerges as a central player in cellular cycle processes, as well as in tumorigenesis. The current studies illuminate the importance of RANBP2 and underscore the need for more extensive studies to validate its clinical applicability in neoplastic interventions. Our review elucidates the significance of RANBP2 across various types of malignancies. Additionally, it delves into exploring RANBP2 as a prospective therapeutic target for cancer treatment, offering insights into the avenues that scholars should pursue in their subsequent research endeavors. Thus, further investigation into RANBP2's role in solid tumorigenesis is eagerly awaited.

## Introduction

Being a widely recognized post-translational protein modification (PTM), small ubiquitin-like modifier modification (SUMOylation) has been garnering growing interest due to its essential role in the cell cycle process.^1^ Research related to SUMOylation dates back to 1995 when scientists initially identified suppressor of mif two 3 (SMT3), the gene responsible for encoding the only SUMO protein in *S. cerevisiae*. This discovery was made through its essential role in centromeric functions and cell division.^2^

Many important cell cycle regulators, including oncogenes or tumorigenesis, are functionally regulated through SUMOylation. Not surprisingly, it is found that SUMO progress is related to the development and metastasis of diverse types of cancers.^3^ In the process of SUMOylation, SUMO E3 ligases facilitate the conjugation by facilitating the proximity of the charged E2 SUMO-conjugating enzyme and the substrates, which is a process of specialization.^4^ In simpler terms, a SUMO E3 ligase takes the activated SUMO from an E2 SUMO-conjugating enzyme and temporarily attaches it in a thioester intermediate state before transferring it to a protein 5. Once the E3 ligase pinpoints the specific target protein, it facilitates the connection between the target and the SUMO-conjugating enzyme (E2), bringing the E2-conjugated SUMO close to the target protein.

RAN binding protein 2 (RANBP2) is a rarely known SUMO E3 ligase and is the vertebrate nuclear pore complex (NPC) protein 6. NPC contributes to the exchange of macromolecules, metabolites, and ions between the nuclear and cytoplasmic areas.^7^, ^8^, ^9^ As a central participant in tumorigenesis and various cellular cycle processes, RANBP2 assumes a pivotal role by serving as a SUMO E3 ligase and associating with NPC. Consequently, disparate expression levels of RANBP2 and mutations within the RANBP2 gene are involved in precipitating distinct forms of malignancies.

## The brief introduction of SUMOylation

PTM represents a common and pivotal mechanism for regulating protein function. Among different types of PTMs, SUMOylation, an enzyme-mediated PTM, biochemically resembles ubiquitination but is functionally distinct from it, which plays essential roles during the cell cycle procedure. The pathway of SUMOylation is mechanistically familiar with ubiquitylation, but SUMO conjugation requires a set of different enzymes.^10^ Conjugation of ubiquitin is initiated by a specific ubiquitin-activating enzyme that catalyzes the formation of ubiquitin adenylate and the subsequent transfer of activated ubiquitin to the thiol site on the ubiquitin adenylate. After that, the ubiquitin ligase is about to catalyze the final ligase reaction.^11^

Currently, only one type of SUMO gene is found in invertebrates, while the process of SUMOylation requires three types of enzymes, namely, E1 SUMO-activating enzyme, E2 SUMO-conjugating enzyme, and SUMO E3 ligase.^12^ Among these enzymes, the E2 SUMO-conjugating enzyme and SUMO E3 ligase are nearly the same, which share 50% amino sequence identity with the E1 SUMO-activating enzyme. A SUMOylation procedure is mediated by the E1 SUMO-activating enzyme, and then activated SUMO forms a complex with the E2 SUMO-conjugating enzyme.^13^^,^^14^ Then, the E2 SUMO-conjugating enzyme-modified molecule can be stimulated by the action of a SUMO E3 ligase.^15^ The SUMO E3 ligases have been assumed to have many abilities, such as binding to E2 SUMO-conjugating enzyme, binding to SUMO moiety via a SUMO-interaction motif domain, and enhancing the transferring process of SUMO from E2 SUMO-conjugating enzyme to substrates.^16^

**Figure 1:**
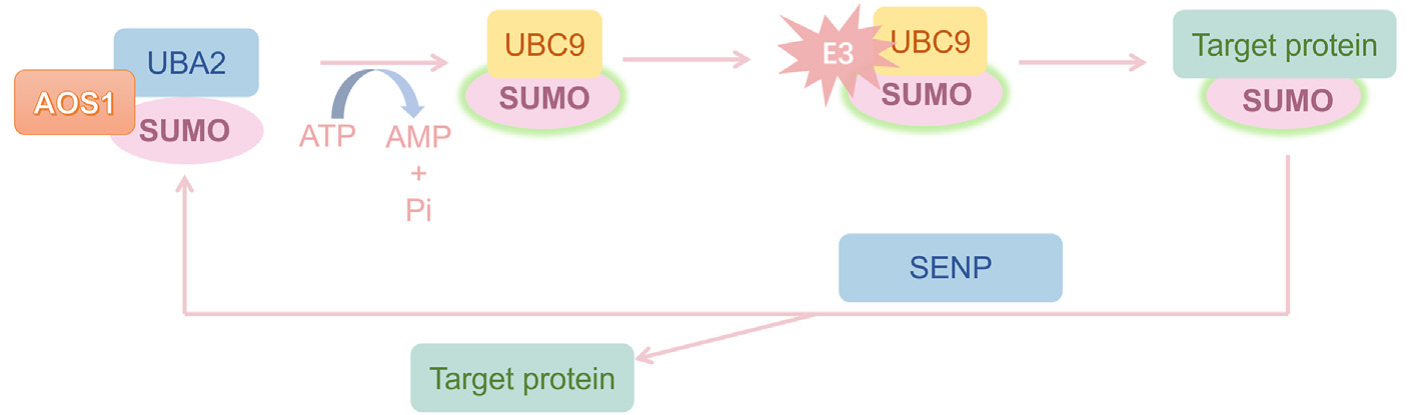
The schematic model of the SUMOylation process. The SUMO protein is activated by the E1 SUMO-activating enzyme UBA2 to form a thioester bond between SUMO and the cysteine residue of AOS1 in an ATP-dependent manner. Then, the activated SUMO is transferred to E2 SUMO-conjugating enzyme. Finally, SUMO E3 ligase enhances the efficiency and specificity of the SUMOylation by mediating the transfer of SUMO from E2 to the target protein by bringing the substrate into proximity. As a reversible process, the SUMOylation can be reversed by SUMO proteases, SENPs, through releasing SUMO from their target protein. UBA2, ubiquitin like modifier activating enzyme 2; AOS1, SUMO activating enzyme subunit 1; SENPs, sentrin-specific peptidases; UBC9, ubiquitin carrier protein 9.

The first enzyme, E1 SUMO-activating enzyme, initiates the process by activating the SUMO protein in an ATP-dependent manner. Subsequently, the activated SUMO is transferred to the E2 SUMO-conjugating enzyme, facilitating the conjugation process. The SUMO E3 ligase plays a crucial role in substrate selection and catalyzes the transfer of SUMO from E2 to the target protein (Fig. 1). However, recent investigations have unveiled a broader spectrum of enzymatic involvement in SUMOylation, expanding beyond the previous enzymes mentioned, as well as other SUMO genes in vertebrates. Four SUMO proteins have been reported in mammals, namely, SUMO-1, SUMO-2, SUMO-3, and SUMO-4. Among them, the sequence of SUMO-2 and SUMO-3 has high similarity, appropriately 97%, which are usually considered as one protein.^16^ Recently, the molecules of SUMOylation add two extra subtypes, named SUMO-4 and SUMO-5. SUMO-4 is associated with type 1 diabetes, which is an artificial gene exhibiting a nucleotide identity of 90% with SUMO-2 gene and an amino acid identity of 87%.^17^ SUMO-5 is a novel SUMO variant located on chromosome 20, which is found to be related to promyelocytic leukemia by increasing polySUMO-2/3 conjugation.^18^

Through the precise cleavage between the C-terminal glycine of SUMO and the substrate lysine, the enzyme responsible for dissociating SUMO from proteins in this process is termed SUMO protease. SUMO proteases exert influence over various cellular processes, and mutations in these enzymes can lead to corresponding diverse defects. The initial identification of a human protein exhibiting as a SUMO-specific protease is sentrin-specific protease 1 (SENP1). Several well-characterized SUMO proteases have been thoroughly elucidated at the biochemical, cellular, and physiological levels.^19^ In certain instances, the mechanisms through which deSUMOylation affects the activity or function of target proteins have also been identified (Table 1).

**Table 1:** The enzymes involved in SUMOylation process.

Types	Genes	Location	Amino acid	UniProt ID	Function
E1 SUMO-activating enzyme	AOS1 (SAE1)	19q13.32	342	Q9UBE0	AOS1 and UBA2 form a heterodimer functioning as a SUMO-activating enzyme for the SUMOylation of proteins
	UBA2 (SAE2)	19q13.11	640	Q9UBT2	
E2 SUMO-conjugating enzyme	UBC9	16p13.3	158	P63279	Accepts SUMO from the ASO1/UBA2 heterodimer and catalyzes their covalent attachment to target proteins with the help of an E3 ligase
E3 SUMO ligase	PIAS1	15q23	651	O75925	Mediates the SUMOylation of their target proteins
	PIAS2	18q21.1	621	O75928	
	PIAS3	1q21.1	628	Q9Y6X2	
	PIAS4	19p13.3	510	Q8N2W9	
	MMS21	8q24.13	247	Q96MF7	
	TRIM19	15q24.1	882	P29590	
	TRIM27	6p22.1	513	P14373	
	TRIM28	19q13.43	835	Q13263	
	TRIM33	1p13.2	1127	Q9UPN9	
	TRIM38	6p22.2	465	O00635	
	RANBP2	2q13	3224	P49792	
	TOPORS	9p21.1	1045	Q9NS56	
SUMO protease	SENP1	12q13.11	644	Q9P0U3	Catalyzes and deconjugates SUMO from target proteins
	SENP2	3q27.2	589	Q9HC62	
	SENP3	17p13	574	Q9H4L4	
	SENP5	3q29	755	Q96HI0	
	SENP6	6q14.1	1112	Q9GZR1	
	SENP7	3q12.3	1050	Q9BQF6	
	SENP8	15q23	212	Q96LD8	

Note: AOS1/SAE1, SUMO activating enzyme subunit 1; UBA2/SAE2, ubiquitin like modifier activating enzyme 2; UBC9, ubiquitin carrier protein 9; PIAS1/2/3/4, protein inhibitor of activated STAT 1/2/3/4; MMS21, methyl methanesulfonate sensitivity gene 21; TRIM19/27/28/33/38, tripartite motif containing 19/27/28/33/38; RANBP2, RAN binding protein 2; TOPORS, TOP1 binding arginine/serine rich protein; SENP1/2/3/5/6/7/8, sentrin-specific peptidase 1/2/3/5/6/7/8.

## RANBP2, SUMO E3 ligase during SUMOylation

**Figure 2:**
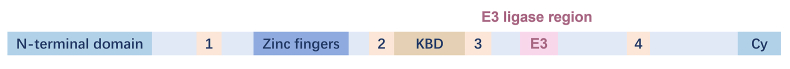
The functional domains of RANBP2 (RAN binding protein 2) protein. RANBP2 protein includes an N-terminal domain, Zinc figure motifs, four RAN-binding domains (light yellow, labeled with No. 1–4), a kinesin-binding domain (KBD), an E3 ligase region, and a cyclophilin-like domain (Cy).

RANBP2 gene is situated on chromosome 2 at the q13 position in the human genome, comprising a total of 29 exons. Since found in 1995, RANBP2 is well known to support a functional role in protein transporting through the NPC.^20^ It constitutes an unusually large protein, with 3224 amino acid residues and an anticipated molecular weight of 358 kDa. It includes an N-terminal domain, Zinc finger motifs, four RAN-binding domains, several FG and XFXFG repeats, a kinesin-binding domain, an E3 domain, and a cyclophilin-like domain.^21^ This protein is a giant scaffold and chimeric procyclin-associated NPC protein associated with the RAN-GTPase cycle.^22^ Located between RAN-binding domain 3 and RAN-binding domain 4, the E3 ligase region comprises an internal repetitive structural domain (IR1-M-IR2), which performs the catalyzing activity of E3 ligase (Fig. 2). Most identified SUMO-binding motifs correspond to SUMO-interaction motifs which are concise protein domains that exhibit selective, non-covalent interactions with SUMO molecules.^23^ It establishes a stable complex NPC with SUMO-modified RanGAP1 (Ran GTPase-activating protein 1) and UBC9 (ubiquitin-conjugating enzyme 9), facilitating E3 ligase enzymatic processes.^24^ Its unique position indicates the existence of mechanisms and functional connections between SUMOylation and nucleocytoplasmic transport.^25^

Small molecules, such as metabolites and ions, can pass through NPC freely, while the diffusion of larger molecules is restricted and governed by their respective size and surface properties which are identified by nuclear transport factor receptors. Subsequently, the complexes undergo sequential interactions with repeat sequences of various nucleoporins, facilitating translocation across NPC and release into the cell nucleus.^26^

As previously elucidated, RANBP2, an important component of NPC, also functions in both mitosis regulation and nucleocytoplasmic transport like SUMO E3 ligase.^27^ The RANBP2 protein demonstrates the capacity to facilitate the SUMOylation of various substrates within mammalian organisms, including but not limited to nuclear receptor subfamily 5 group A member 2 (NR5A2) and β-arrestin 2.^28^^,^^29^ The heterogeneity of substrates targeted by RANBP2 may elucidate its involvement in numerous cellular processes associated with RANBP2, including nuclear transport, gene expression, chromosome segregation during mitosis, chromatin regulation, and even antiviral activity.^20^ Mutations in the non-E3 domains of RANBP2 are found to be associated with heightened susceptibility to acute necrotizing encephalopathy, suggesting that mutations in RANBP2 elicit cerebral pathology only under specific circumstances.^30^ In addition, extant research posits an association between RANBP2 and mitochondrial transport and functionality. Anomalous expression of the driving protein-binding domain of RANBP2 induces perinuclear aggregation of mitochondria within cells and compromises mitochondrial membrane potential.^31^ PRKN (parkin RBR E3 ubiquitin protein ligase) gene is SUMOylated and selectively associated with RANBP2, culminating in the manifestation of a distinct familial form of Parkinson's disease.^32^ Numerous investigations underscore the pivotal role of RANBP2 in viral infections,^30^ wherein RANBP2 exhibits the capacity to actively facilitate the nuclear entry or exit of nuclear viruses. Furthermore, empirical evidence supports the assertion that RANBP2 can promote the decapitating process of specific viruses and participate in cellular anti-defense mechanisms, thereby suppressing viral replication.^33^ This multifaceted involvement of RANBP2 in various stages of viral infection underscores its significance as a key mediator in the intricate interplay between viruses and host cells.^34^ However, although the UbiBrower database provides a comprehensive repository of known and predicted ubiquitin ligase and deubiquitinase–substrate interactions,^35^ there is currently no exact substrate enlisted for RANBP2. Table 2 summarizes the reported substrates of RANBP2 as SUMO E3 ligase in malignancies.

**Table 2:** The substrates of RANBP2 as SUMO E3 ligase in malignancies.

Substrates	Cancer type	Mechanism	Reference
LASP1	Hepatocellular carcinoma	Increasing the expression of HER2	47
NR5A2	Hepatocellular carcinoma	Decreasing AFP level	53
CEBPα	Hepatocellular carcinoma	Activating RANBP2-CEBPα-OGA pathway	29,56
SIRT1	Hepatocellular carcinoma	Promoting the degradation of FTO	58
IL-33	Hepatocellular carcinoma	Inhibition of IRF1 transcription	59
p27kip1	Cholangiocarcinoma	Accumulation of p27kip1 in nucleus	69
DAXX	Gastric cancer	Nuclear translocation	74
β-arrestin 2	Breast cancer	Promoting nuclear translocation of MDM2	28
TCF4	Cervical cancer	Activating the Wnt/β-catenin signaling pathway	86
p53	Prostate cancer	Promoting nuclear output	98

Note: RANBP2, RAN binding protein 2; HER2, human epidermal growth factor receptor 2; AFP, alpha fetoprotein; CEBPα, CCAAT/enhancer-binding protein alpha; OGA, O-GlcNAcase; FTO, fat mass and obesity-associated protein; IRF1, interferon regulatory factor 1; LASP1, LIM and SH3 domain protein; NR5A2, nuclear receptor subfamily 5 group A member 2; CEBPα, enhancer-binding protein alpha; SIRT1, NAD-dependent deacetylase sirtuin-1; IL-33, interleukin 33; p27kip1, cyclin-dependent kinase inhibitor 1B; DAXX, death domain-associated protein; TCF4, transcription factor 4; p53, tumor suppressor protein.

## The involvement of RANBP2 and its related SUMOylation in malignancies

### Hepatocellular carcinoma (HCC)

As one of the leading causes of cancer-related death, HCC is an aggressive cancer with limited therapeutic options.^36^ Given that great achievements related to diagnosis and therapy have been gained, the clinical outcome of patients with HCC remains unsatisfactory owing to treatment resistance, tumor metastasis, and/or recurrence.^37^^,^^38^ It is thus that searching the HCC carcinogenesis at the molecular level and identifying targets for developing efficacious therapeutics brook no delay.^39^

Although the risk factors are diverse for HCC oncogenesis, hepatitis B virus (HBV) infection is one of the important pre-cancerous diseases. Chronic viral hepatitis creates a microenvironment in the liver characterized by a cycle of cell death and regeneration, favoring the growth of hepatocytes with proliferative advantages, ultimately leading to cancer.^40^ Direct mechanisms involve the transformation of viral proteins, such as the well-known HBx or deleted Pre-S, 8, 9, and 10 proteins.^41^ Comparative analysis between cohorts of individuals lacking HBV and those expressing the hepatitis B surface antigen reveals a notable distinction. In the demographic spanning both genders and the age range of 30–75 years, the lifetime cumulative incidence rates of liver cancer stand at 27.38% for hepatitis B surface antigen-positive males and 7.99% for their female counterparts.^42^ This observation underscores the profound impact of HBV infection on the development of HCC. Among the molecules encoded by HBV, HBV X supermolecule (HBx) interferes with molecules that are related to the proliferation and apoptosis of HCC cells.^43^ LIM and SH3 domain protein 1 (LASP1) is elucidated as a multi-functional protein that is correlated with the grade, size, and metastasis of tumors in clinical samples.^44^ It is shown that ectopic expression of HBx enhanced the expression of LASP1, which promoted the proliferation and migration of HCC cells.^45^ However, RANBP2 was identified as an interacting partner with LASP1, facilitating the SUMOylation of LASP1, thereby leading to the up-regulation of human epidermal growth factor receptor 2 (HER2) expression in hepatoma cells, which is usually up-regulated in HCC as a key signaling molecule and potential therapeutic target.^46^^,^^47^ There is no doubt that the detailed mechanisms indicated to HBx, SUMOylated by RANBP2, related to the increase in HER2 offer opportunities for the development of targeted therapies aimed at mitigating the aggressive nature of HCC.

Alpha fetoprotein (AFP) is a well-known biomarker for HCC diagnosis and monitoring, initially identified by Bergstrand and Czar in 1956 within human fetal serum.^48^ Besides the regular PTM procedure, epigenetic regulation like methylation, and acetylation status also influence the expression of AFP.^49^^,^^50^ NR5A2, alternatively referred to as liver receptor homolog 1 (LRH-1), exhibits high expression levels in both the liver and intestine.^51^ Furthermore, AFP gene is located between the albumin and alpha-fetoprotein genes, which are activated by NR5A2.^52^ The stability of NR5A2 is determined by gp96, one of the heat shock proteins.^53^ Interestingly, it is confirmed that RANBP2 shares certain binding sites with gp96, which may result in an enhanced level of SUMOylation of NR5A2 when gp96 is depleted.^53^ Collectively, the findings provide evidence of the interaction between cellular gp96 and its client protein, NR5A2, which exerts a specific inhibitory effect on the binding of NR5A2 to RANBP2, and subsequently impedes the RANBP2-mediated SUMOylation of NR5A2. Therefore, when NR5A2 is not SUMOylated, the expression level of AFP naturally increases accordingly.^53^

In addition to its reliance on AFP as a pivotal biomarker, HCC encompasses an intricate landscape of molecular alterations. O-linked β-N-acetylglucosaminylation (O-GlcNAcylation) is a highly prevalent and dynamic PTM in multicellular organisms. It is a unique type of glycosylation whereby a single sugar moiety, O-linked N-acetylglucosamine (O-GlcNAc), is typically transferred to the hydroxyl groups of serine and threonine residues of proteins.^54^ By delving into the mechanics of O-GlcNAcylation, it is found that O-GlcNAc's cycling is only conducted by one “writer” for protein O-GlcNAcylation, named O-GlcNAc transferase (OGT), and one “eraser”, called O-GlcNAcase (OGA).^55^ The promoter region of the OGA gene contains two binding sites of transcriptional factor CCAAT/enhancer-binding protein alpha (CEBPα). It is reported that elevated protein levels of OGA and a concomitant decrease in O-GlcNAc level were observed upon the overexpression of CEBPα.^56^ In the meantime, RANBP2 might inhibit the transcriptional activity of CEBPα through both NPC and SUMOylation procedures.^56^ Thus, pharmacological interventions targeting the RANBP2-CEBPα-OGA pathway may signify a burgeoning therapeutic strategy for HCC.

During the progression and metastasis of HCC, numerous proteins exhibit altered functionality because of SUMOylation. N^6^-methyladenosine (m^6^A) plays a pivotal role in expediting pre-mRNA processing and facilitating mRNA transportation within mammals. The dynamic of m^6^A is harmonized by adenosine methyltransferases (“writers”), m^6^A-binding proteins (“readers”), and demethylases (“erasers”). Among them, fat mass and obesity-associated protein (FTO), as an eraser, participates in the reversible methylation of m^6^A.^57^ FTO, as a m6A demethylase, is detected to be a decisive factor in the fate of sirtuin 1 (SIRT1)-induced HCC oncogenesis.^58^ In the context of HCC, the precise complexity of SIRT1 regulation in m^6^A modification is achieved primarily through the attenuating effects of SIRT1 on HCC tumorigenicity by FTO. This is because FTO undergoes SIRT1-mediated down-regulation through its deacetylase function. Notably, SIRT1 can activate the activity of RANBP2 and increase its stability, resulting in reduced FTO intensity by activating RANBP2 as SUMO E3 ligase. Importantly, in the condition of RANBP2 depletion, the activated SIRT1 does not reduce FTO intensity. So, SIRT1 inhibits HCC development by attenuating the mRNA expression of the tumor suppressor gene GNAO1 (G protein subunit alpha O1) through FTO-dependent m^6^A modification.^58^

In addition, it is imperative to underscore the significance of HCC resulting from immune system evasion. High expression of interleukin 33 (IL-33) was proved to be related to the suppressive of HCC, while RANBP2 SUMOylated IL-33 has a role in tumorigenesis.^59^ The SUMOylation by RANBP2 acidifies nuclear factor IL-33, thereby disrupting the ubiquitin-mediated protein degradation of interferon regulatory factor 1 (IRF1) and transcriptionally enhancing the expression of programmed death receptor ligand 1 (PD-L1), an immune-supporting factor in the tumor microenvironment. In addition, SUMOylated nuclear IL-33 can promote the production of interleukin 8 (IL-8), which stimulates the IL-8 signaling pathway in macrophages, thereby promoting the migration and polarization of macrophages to the tumor-permissible phenotype. Overall, it is warranted to unravel the intricate mechanisms through which IL-33 exerts its effects in HCC, ultimately offering new insights for the development of therapeutic strategies targeting this cytokine in the context of HCC.

**Figure 3:**
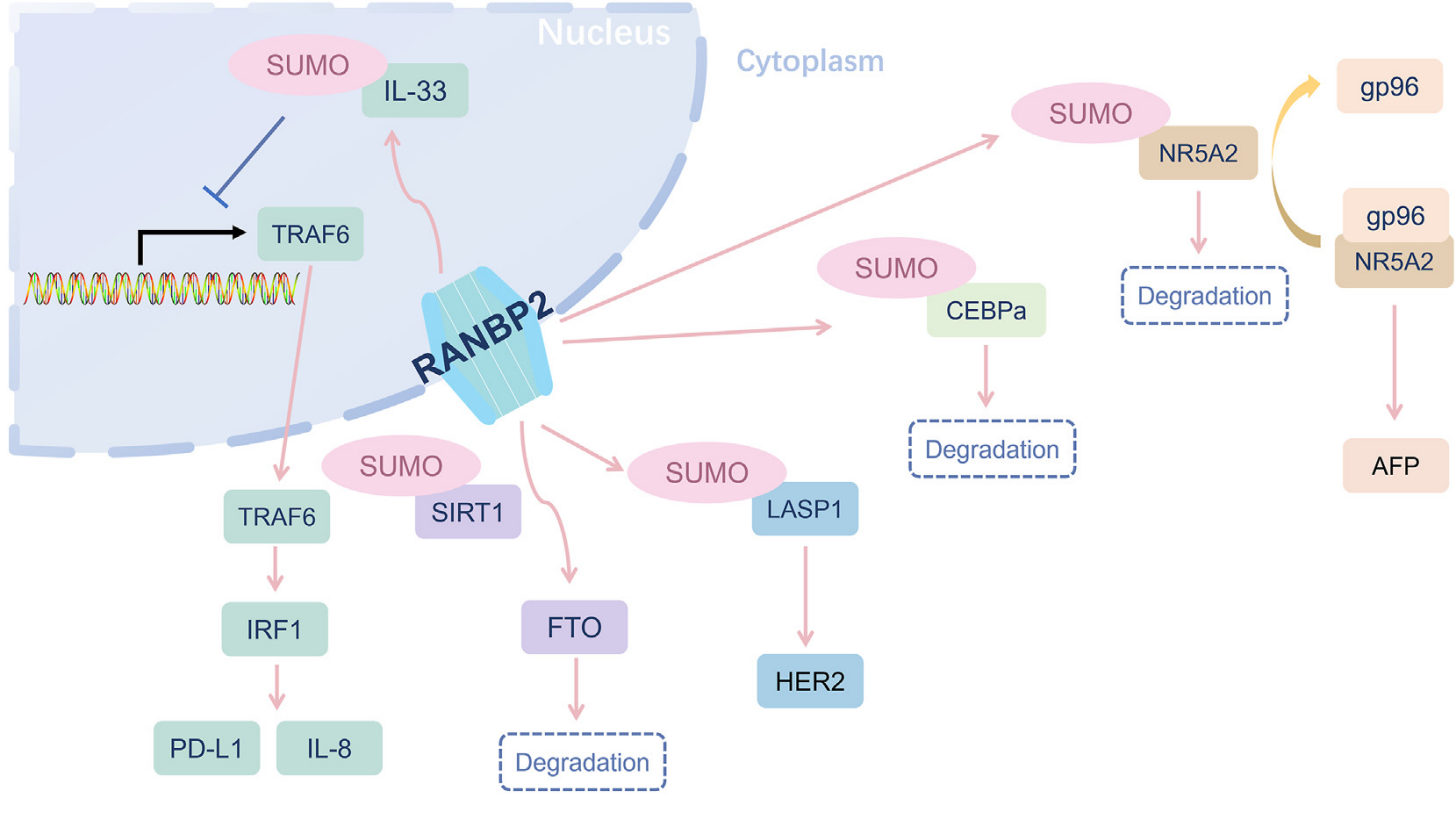
The molecular mechanism of RANBP2 in the development of hepatocellular carcinoma. RANBP2, RAN binding protein 2; IL-33, interleukin 33; IL-8, interleukin 8; TRAF6, TNF receptor associated factor 6; IRF1, interferon regulatory factor 1; PD-L1, programmed death receptor ligand 1; SIRT1, sirtuin 1; FTO, fat mass and obesity-associated protein; LASP1, LIM and SH3 domain protein; HER2, human epidermal growth factor receptor 2; CEBPα, enhancer-binding protein alpha; NR5A2, nuclear receptor subfamily 5 group A member 2; AFP, alpha fetoprotein.

In summary, RANBP2 contributes importantly to the mechanism of HCC cell growth via prevalent post-translation modification SUMOylation. This modification plays an important role in tumor cell proliferation, migration, and invasion, as well as immune escape (Fig. 3).

### Cholangiocarcinoma

Cholangiocarcinoma, originating from malignant transformations of epithelial cells at various loci within the biliary tree, represents a formidable clinical challenge.^60^ Although surgical intervention stands as the primary therapeutic modality for biliary tract cancer, the associated 5-year survival rates remain dishearteningly low.^61^ Chemotherapeutic regimens involving gemcitabine and cisplatin are frequently employed in cases where surgical options are precluded, yet they yield a median overall survival of less than one year.^62^

Cyclin-dependent kinase inhibitor 1B (CDKN1B), also known as p27kip1, serves as a crucial cell cycle regulator by inhibiting cyclin-dependent kinases, particularly during the transition from the G1 to S phase.^63^ The suppression of nuclear p27kip1 expression plays a pivotal role in governing cell proliferation and the malignant progression of tumors.^64^ Due to its functional impairment and aberrant subcellular localization, p27kip1 has emerged as a tumor suppressor gene with significant implications in the treatment of various cancers, including breast cancer,^65^ pancreatic cancer,^66^ and colorectal cancer.^67^ In the context of cholangiocarcinoma, it has been demonstrated that p27kip1 undergoes SUMOylation, a process mediated by upstream molecules such as ubiquitin-conjugating enzyme E2 I (UBE2I)^64^ and RING finger protein (RNF4),^68^ influencing the tumorigenic processes. The inhibition of cholangiocarcinoma cell proliferation by p27kip1 can be attenuated by overexpression of RANBP2, which was attributed to the fact that SUMOylation of p27kip1 facilitates the nucleoplasmic translocation of p27kip1 and attenuates the G1 arrest brought about by the accumulation of p27kip1 in the nucleus.^69^ To sum up, the SUMOylation of p27kip1, facilitated by RANBP2, fosters the proliferation of cholangiocarcinoma cells, which may serve as a potentially potent therapeutic target in the eradication of cholangiocarcinoma development and relapses.

### Gastric cancer

Gastric cancer, encompassing both cardia and non-cardia gastric cancers, remains a significant global malignancy. The American Cancer Society's estimates for gastric cancer in the United States for 2024 are about 10,880 deaths (6490 men and 4390 women).^70^ This grim statistic translates to approximately one in every twelve global deaths, positioning gastric cancer as the fifth most frequently diagnosed malignancy and the third leading cause of cancer-related mortality. The scale of its impact underscores the need for comprehensive research initiatives and innovative strategies aimed at prevention, early detection, and treatment modalities.^71^ Death domain-associated protein (DAXX) is a death domain-associated protein intricately linked to apoptotic, anti-apoptotic, and transcriptional regulatory processes. It is found that DAXX is uniformly expressed throughout the body, except in the testes and thymus.^72^ Previous investigations have suggested that DAXX may exhibit distinct functionalities with its diverse subnuclear compartments.^73^ Intriguingly, DAXX exhibits opposing subcellular localizations within the nucleus and cytoplasm, highlighting its versatile roles. The expression levels of cytoplasmic DAXX have been found to correlate with enhanced survival outcomes, while elevated levels of nuclear DAXX indicate a poorer prognosis in gastric cancer.^73^ Although there are no discernible differences in mRNA levels of DAXX across all cellular compartments, variations exist at the protein level. The interaction of DAXX with RANBP2 may assemble UBC9 and DAXX to form SUMOylation complexes, triggering its translocation from the cytoplasm to the nucleus, underscoring its pivotal role in cellular dynamics,^74^ which promotes the proliferation of gastric cancer cells. The implication of DAXX in tumorigenesis is evident, underscoring the significance of mRNA-level dysregulation of DAXX as a potential therapeutic target.

### Breast cancer

The incidence of breast cancer has become the top type of malignancy in females. In the United States in 2024, it is projected that approximately 310,720 new cases of invasive breast cancer will be diagnosed in women, along with 2790 cases in men. Additionally, there will be an anticipated 56,500 diagnoses of ductal carcinoma *in situ* exclusively in women.^70^ These estimations highlight the continued significance of breast cancer as a prevalent health concern, warranting ongoing attention and efforts in research, prevention, and treatment strategies.

The tumor suppressor p53 diligently safeguards genomic integrity, thereby thwarting the initiation of tumorigenesis through its multifaceted role in orchestrating cellular responses.^75^ It achieves this mission by overseeing crucial processes, such as cell cycle arrest, DNA repair, induction of senescence, and initiation of programmed cell death, while simultaneously exerting influence over autophagic mechanisms and the metabolic pathways relevant to oncogenesis.^76^ It is confirmed that MDM2 is elucidated to function as an antagonist of p53.^77^ Originally unearthed for their pivotal roles in G protein-coupled receptor (GPCR) regulation, β-arrestins have since demonstrated their capacity as central scaffolding hubs governing a plethora of signaling pathways.^78^ It is substantiated that the translocation of MDM2, a principal suppressor of p53, from the nuclear compartment to the cytoplasm engenders elevated p53 functionality.^79^ However, the non-covalent interaction of the β-arrestin 2 with a SUMOylated protein partner may contribute to nucleoplasm transport rather than SUMOylated β-arrestin 2.^28^ SUMO-conjugated proteins rely on direct non-covalent protein–protein interactions between the covalent-bound SUMO and proteins containing SUMO binding domains.^80^ Nuclear input of β-arrestin 2 after SUMOylation is inhibited, and thus its ability to delocalize MDM2 from the nucleus to the cytoplasm is diminished, thereby enhancing p53 signaling and suppressing tumorigenesis in breast tumor cells. In the context of this modification, the mutation in β-arrestin 2 hinders the nuclear translocation of MDM2, leading to its sequestration in the cytoplasm, and impairing its ability to augment the p53 signaling pathway.^24^

Besides SUMOylation, RANBP2 also plays the role of E3 ligase in the sort of familiar PTM, relevant to breast cancer as well. Neddylation is a reversible covalent process involving the conjugation of a ubiquitin-like molecule NEDD8 (neuronal precursor cell-expressed developmentally down-regulated protein 8) to a specific lysine residue situated within the substrate protein,^81^,^82^ which is familiar with SUMOylation. The neddylation system suppresses the basal c-Jun NH2-terminal protein kinase (JNK) phosphorylation in breast cancer cells. Augmented basal JNK phosphorylation impedes the proliferation and the epithelial-to-mesenchymal transition of breast cancer cells.^83^ In the neddylation procedure, RANBP2 induces the neddylation of mitogen-activated protein kinase kinase 7 (MKK7), which activates JNK working as a sequential protein. Thus, the neddylation process inhibits the JNK pathway by the basal kinase activity of MKK7 in breast cancer cells, elevating tumorigenesis.

### Cervical cancer

Cervical cancer stands as one of the prevailing malignancies, afflicting the female population, with a global mortality rate ranking fourth in its grim significance.^84^ The escalating incidence and mortality rates of cervical cancer in China represent a serious public health concern, imperiling the well-being of the female population.^85^

The nucleolar and spindle-associated protein (NUSAP) has recently emerged as a microtubule and chromatin-binding protein in vertebrates, functioning prominently during the interphase of cell division.^86^ Depletion of NUSAP results in aberrant spindle formation and erroneous chromosome segregation, and ultimately impedes cellular proliferation. Nucleolar and spindle associated protein 1 (NUSAP1), through its interaction with RANBP2, induces the acetylation of transcription factor 4 (TCF4) and subsequently augments the activity of the Wnt/β-catenin signaling pathway.^86^ The Wnt/β-catenin signaling pathway plays a pivotal role in regulating migratory capabilities and remains persistently activated in cervical cancer, thereby influencing the progression of malignancies. This sustained activation significantly impacts crucial aspects of cancer, such as epithelial-to-mesenchymal transition and cancer stem cell characteristics, both of which contribute substantially to the metastatic potential of a spectrum of malignancies.^87^^,^^88^ Guided by NUSAP1, TCF4 after SUMOylation significantly activated the Wnt/β-catenin signaling pathway, thereby promoting cervical cancer tumorigenesis and metastasis.

As mentioned before, m^6^A is a common physiological modification in eukaryotes, also involved in tumorigenesis. One of the initial protein families identified and characterized is the YTH domain-containing protein family (YTHDF), comprising YTHDF1, YTHDF2, and YTHDF3. These proteins are known to be predominantly localized within the cellular cytoplasm.^89^, ^90^, ^91^, ^92^ It is demonstrated that the m^6^A reader YTHDF1 is highly expressed in cervical cancer and is related to the poor prognosis of cervical cancer patients.^93^ RANBP2 promoted the growth, migration, and invasion of cervical cancer cells, while the correlation analysis revealed a positive association between the protein expression of RANBP2 and YTHDF1 in cervical cancer, collectively indicating the noteworthy involvement of the YTHDF1-m^6^A-RANBP2 axis in cervical cancer. Specifically, the up-regulation of RANBP2 expression induced by YTHDF1 potentially augments RAN-GTPase activity, thereby exacerbating the progression of cervical cancer. This complex interaction warrants further investigation in future studies. Consequently, YTHDF1 emerges as a promising therapeutic target for cervical cancer treatment.^93^

### Prostate cancer

Prostate cancer exhibits a notable disparity in 5-year relative survival rates based on disease stage. For most men diagnosed with localized- or regional-stage prostate cancer, the 5-year survival rate approaches nearly 100%, reflecting a favorable prognosis.^70^ However, this rate sharply declines to 34% for individuals diagnosed with distant-stage disease, emphasizing the critical impact of disease staging on prognosis. These statistics underscore the significance of early detection and intervention in prostate cancer, as timely diagnosis significantly contributes to improved survival outcomes. Novel biomarkers are needed to enhance clinical outcomes and tailor personalized treatments.^94^

**Figure 4:**
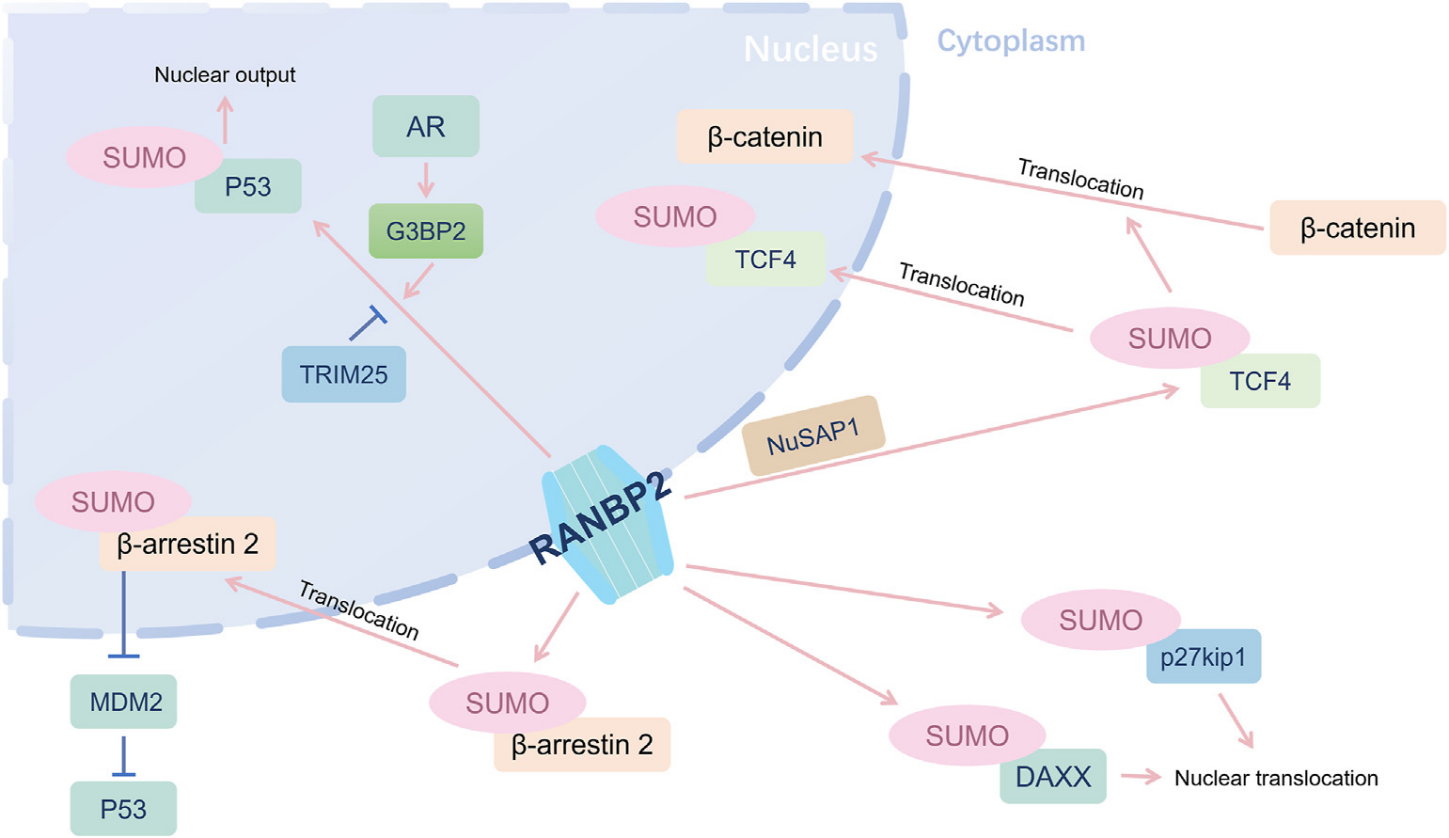
The reported substrates of RANBP2 in other solid malignancies besides hepatocellular carcinoma. RANBP2, RAN binding protein 2; AR, androgen receptor; G3BP2, GTPase-activating protein-binding protein 2; TRIM25, tripartite motif containing 25; TCF4, transcription factor 4; NUSAP, nucleolar and spindle-associated protein; p27kip1, cyclin-dependent kinase inhibitor 1B; DAXX, death domain-associated protein.

Androgen receptor serves as a transcription factor, planning the differentiation of the prostatic epithelium by modulating the expression of hundreds of genes, as well as assuming a vital role in the progression of prostate cancer.^95^ p53 acts as a pivotal node in tissue cells, orchestrating responses to various types and levels of stress through mechanisms such as apoptosis, cell cycle arrest, senescence, DNA repair, cellular metabolism, and autophagy.^96^ As mentioned above, dysfunction of p53 contributes to malignant transformation, implicated in facilitating malignant transformation. It is elucidated that GTPase-activating protein-binding protein 2 (G3BP2) plays the regulatory role in modulating androgen-mediated p53 nuclear output through the RANBP2-mediated SUMOylation.^97^ The nuclear export of p53 is mediated by RANBP2-mediated SUMOylation regulated by G3BP2, which involves androgen receptor in this process. Plus, the investigative findings illuminate a novel androgen receptor-mediated mechanism of p53 suppression, thereby fostering cellular proliferation, migration, and therapeutic resistance in prostate cancers.^98^ Tripartite motif-containing 25 (TRIM25), identified as a key target gene of estrogen receptor alpha, serves as an integral component in the negative regulation of p53 activity. Their interaction with G3BP2 further underscores their involvement in this regulatory cascade.^99^ G3BP2 has been demonstrated to induce tumor growth and an augmentation of nuclear p53 accumulation. Diminishing TRIM25 attenuates the SUMOylation of p53 by RANBP2, thereby suppressing tumorigenesis, which posites TRIM25 as a prospective therapeutic target. In summary, the impact of RANBP2 on the SUMOylation of p53 is found in lung cancer, which diminishes the regulatory control exerted by tumor suppressor factors over cellular apoptosis, thereby culminating in tumorigenesis (Fig. 4).

### Other types of solid malignancies

Besides the solid malignancies mentioned above, the investigation of RANBP2 was also conducted in other types of cancers through bioinformatics methods. In glioblastoma multiforme, the most malignant intracranial neoplasm, originating from glial cells,^100^, ^101^, ^102^ Li et al scrutinized the SUMOylation regulatory factors and discerned that UBE2I, ubiquitin like modifier activating enzyme 2 (UBA2), protein inhibitor of activated STAT 3 (PIAS3), and SENP1 exhibit heightened expression levels in glioblastoma multiforme, while conversely, PIAS1, RANBP2, SENP5, and SENP2 manifested down-regulation in this context. Based on their findings, SUMOylation regulatory factors were ascertained to potentially participate in cellular processes, such as the cell cycle and DNA replication in glioblastoma multiforme.^103^ For oral cancer, one of the most formidable challenges confronting humanity,^104^ missense mutations, single nucleotide polymorphisms, and C > T transitions emerge as the prevailing single nucleotide variation categories, with RANBP2 and SENP6 exhibiting the highest single nucleotide variation frequencies, among 15 SUMOylation regulatory factors examined in 323 cases of oral squamous cell carcinoma with an additional 32 cases representing normal tissue, as well as 96 cases of single nucleotide variations of oral squamous cell carcinoma. Discrepancies in the expression patterns of SUMOylation regulatory factors underscore the potentially pivotal role of SUMOylation in the pathogenesis of oral squamous cell carcinoma.^105^ However, such results need further *in vitro* and *in vivo* verification.

## The other molecular mechanism of RANBP2 involved in malignancies

Due to the pivotal role played by nuclear pore proteins in nuclear processes, such as chromatin silencing, transcriptional regulation, and DNA damage repair, the occurrence of numerous types of tumors is intricately linked to these proteins. However, only a limited subset of NPC is associated with tumorigenesis, and RANBP2 stands out as one such exemplar.^106^

Colorectal cancer represents a profoundly intricate and heterogeneous disease. Recent technological advancements over the past few years have facilitated a more in-depth analysis of the genetic and epigenetic characteristics of colorectal tumors, leading to the emergence of diverse molecular classifications.^107^ The protein kinase B-Raf proto-oncogene (BRAF), situated downstream of RAS within the RAS-RAF-MEK-ERK kinase cascade, assumes a pivotal role in cellular signal transduction, manifests itself in approximately 8%–10% of patients diagnosed with colorectal cancer.^108^ Tumors manifesting the genetic hallmark under consideration are denominated as “BRAF-like”, exhibiting an analogous unfavorable prognosis irrespective of the occurrence of the BRAF mutation.^109^ In BRAF-like colorectal cancer cells, the suppression of RANBP2-induced cell death is propelled by mitotic defects. In mechanistic terms, the attenuation of RANBP2 has been observed to diminish the microtubule growth of centrosomes, thereby inducing spindle perturbations. This phenomenon is postulated as a plausible causative factor underlying the induction of apoptotic demise in BRAF-like colorectal cancer cells. RANBP2 assumes a crucial role in the dynamics of the mitotic process, with its loss triggering spindle disturbances and instigating a spectrum of mitotic defects that culminate in cellular demise.^110^ In summary, RANBP2 mediates apoptosis in BRAF-like colorectal cancer cells by spindling apparatus movement during mitosis.

Lung cancer remains a pervasive global challenge, resulting in a greater number of fatalities among men than any other cancer worldwide. Targeted biologics and immunomodulators have improved therapeutic efficacy, but additional treatments, potentially in combination with biologics, are needed to further improve patient survival.^111^ RANBP2 has been documented as a multifaceted protein, intricately intertwined with crucial cellular processes encompassing nucleocytoplasmic transport and mitosis. Specifically, RANBP2 serves as a vital component in keeping stability during the intricate choreography of mitosis. It accomplishes this by orchestrating the precise recruitment of DNA Topoisomerase II (TopoII) to centromeric regions, a pivotal event that underpins the integrity of the genetic material as it undergoes partitioning.^27^ TopoII functions as a pivotal entity in mediating chromatin monomer integration during mitosis, while whether RANBP2 plays a role as an E3 ligase in the SUMOylation of TopoII within its SUMOylation class requires further investigation.^25^ This is essential due to the observed lack of correlation between the chemical sensitivity of amrubicin, a fully synthetic anthracycline anti-cancer agent and effective TopoII inhibitor, and the mRNA expression levels of RANBP2, TopoII-α, and TopoII-β genes. Further research is needed to elucidate the potential involvement of RANBP2 as an E3 ligase in the SUMOylation of TopoII and its implications in chemosensitivity.^112^ These activities hold paramount significance in the onset and progression of small-cell lung carcinoma. Thus, additional research remains requisite in the realm that remains void, awaiting further investigation and exploration.

## Perspectives in clinics

RANBP2, a key player in nucleocytoplasmic transport, has garnered significant attention as a potential SUMOylation ligase. Recent studies highlight its role in the regulation of NPC assembly and disassembly, impacting nucleocytoplasmic transport dynamics. Among all the research about RANBP2, understanding the functional consequences of RANBP2-mediated SUMOylation is a central focus. Nevertheless, unraveling the precise substrate specificity of RANBP2 is a challenge. Identifying key protein targets and elucidating the molecular mechanisms underlying their SUMOylation by RANBP2 remains an active area of investigation.

Challenges are specifically expressed as the intricate interplay between RANBP2 and other SUMOylation regulators, deciphering the complex regulatory networks governing RANBP2 activity is crucial for a comprehensive understanding of its role in cellular processes. Obtaining detailed information about RANBP2 and its interactions with substrates is challenging as well. Advances in structural biology techniques are needed to provide detailed insights into the molecular basis of RANBP2-mediated SUMOylation.

Current research is full of opportunities and challenges, and there are still gaps to be filled. For example, although it is known from previous studies that RANBP2, as a SUMO E3 ligase, has been implicated in the development of a variety of cancers, the existence of corresponding inhibitors of RANBP2 that can inhibit its role as an E3 ligase remains a mystery. Despite tumorigenesis, the specific role of RANBP2-mediated SUMOylation in various disease contexts remains understudied. Investigating its involvement in diseases such as neurodegeneration and viral infections is essential for potential therapeutic interventions. The limited availability of robust *in vivo* models that mimic RANBP2-related SUMOylation events hinders progress. Developing relevant model systems will enhance our ability to study the physiological relevance of RANBP2 in a complex cellular environment.

Above all, the potential clinical applications of RANBP2 as a therapeutic target are intriguing. Modulating RANBP2 activity may offer novel avenues for intervention in diseases where dysregulated SUMOylation contributes to pathogenesis. However, translating basic research findings into clinical applications requires a deeper understanding of the molecular mechanisms involved. Addressing the current challenges and research gaps will undoubtedly contribute to unlocking the full spectrum of RANBP2-mediated SUMOylation and its implications in health and disease.

## Conclusion

Since discovered as a pivotal nuclear pore protein and a SUMOylation E3 ligase, RANBP2 has garnered considerable attention. Initially, there was a prevailing consensus that RANBP2 primarily exerted its influence on tumorigenesis by serving as a nuclear pore protein, modulating the ingress and egress of substances to and from the cell nucleus.

Recent studies have identified several substrates of RANBP2-mediated SUMOylation and provided insights into its regulatory mechanisms. However, a comprehensive understanding of its functional diversity and significance in different cellular contexts still needs further studies. Noteworthy is the observation that the investigations on RANBP2 primarily reside within the confines of laboratory experimentation. The substantiation of tumorigenic occurrences predominantly subsists at the cellular level. This implicitly underscores the imperative that while RANBP2 emerges as a prospective therapeutic target for neoplastic interventions, its translation into clinical validation is a gap. More significantly, its application in patient care mandates a requisite for more extensive and profound investigations. The current body of research, predominantly circumscribed to experimental settings and cellular phenomena, accentuates the essentiality and profound significance of conducting further in-depth studies to ascertain the veracity and translational potential of RANBP2 as an efficacious therapeutic target in the clinical milieu.

## Funding

This work was supported by the 10.13039/501100001809National Natural Science Foundation of China (No. 82273457), the Guangdong Basic and Applied Basic Research Foundation of China (No. 2023A1515012762 and 2021A1515010846), Special Grant for Key Area Programs of Guangdong Education Department (China) (No. 2021ZDZX2040), Science and Technology Special Project of Guangdong Province, China (No. 210715216902829), and “Dengfeng Project” for the construction of high-level hospitals in Guangdong Province—First Affiliated Hospital of Shantou University College Supporting Funding (No. 202003-10).

## Conflict of interests

The authors declared no conflict of interests.
